# Causes of hypercapnic respiratory failure and associated in‐hospital mortality

**DOI:** 10.1111/resp.14388

**Published:** 2022-10-09

**Authors:** Yewon Chung, Frances L. Garden, Guy B. Marks, Hima Vedam

**Affiliations:** ^1^ South Western Sydney Clinical School UNSW Medicine Sydney New South Wales Australia; ^2^ Ingham Institute for Applied Medical Research Liverpool New South Wales Australia; ^3^ Department of Respiratory and Sleep Medicine Liverpool Hospital Liverpool New South Wales Australia

**Keywords:** epidemiology, hypercapnia causes, respiratory failure, respiratory insufficiency, type 2

## Abstract

**Background and Objective:**

Hypercapnic respiratory failure (HRF) can occur due to severe respiratory disease but also because of multiple coexistent causes. There are few data on the prevalence of antecedent causes for HRF and the effect of these causes on prognosis, especially where study inclusion has not been biased with respect to primary diagnosis, interventions received or clinical outcome. We sought to determine the prevalence of pre‐specified conditions among patients with HRF and to determine the effect of these causes on in‐hospital mortality.

**Methods:**

Cross‐sectional study of patients with HRF from 2013 to 2017. Inclusion criteria were PaCO_2_ >45 mm Hg and pH ≤7.45. Causes of interest were identified using diagnosis codes from hospital records. We used directed acyclic graphs to inform logistic regression models for the outcome of in‐hospital death.

**Results:**

We identified 873 persons with HRF in the study period. Mean (SD) age was 69 years and 50.4% were males. Acidosis (pH <7.35) was present in 488 (55%) cases. Most (83%) had one or more of the following: obstructive lung disease, lower respiratory tract infection, congestive cardiac failure, sleep disordered breathing, neuromuscular disease, opioid or benzodiazepine use. In‐hospital mortality was 12.8%. Obstructive lung disease and cardiac failure were associated with a lower risk of death, whereas respiratory tract infection and neuromuscular disease were associated with increased risk of death.

**Conclusion:**

HRF is associated with a range of potentially causative conditions, which significantly impact hospital survival. Systematic evaluation of patients with HRF may increase detection of treatable comorbidities.

## Graphical abstract

This is the first study of causes of hypercapnic respiratory failure at a population level. Obstructive lung disease and congestive cardiac failure were highly prevalent. The underlying cause or causes impacted in‐hospital survival. Clinicians must consider systematic evaluation of patients with hypercapnia to detect potentially treatable conditions.

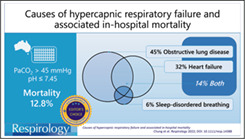

**See related** Editorial

## INTRODUCTION

Hypercapnic respiratory failure (HRF) is a physiological endpoint with multiple potential causes. While individual causes of HRF warrant disease‐specific treatments, most patients with HRF require hospitalization and many need ventilatory support in a dedicated critical care ward, invoking considerable healthcare costs. Hence, even though HRF can occur due to many diseases, it can also be viewed as a single, albeit heterogeneous, condition that constitutes a substantial problem for acute health facilities in many countries.

To date, there has been no systematic evaluation of contributing causes of HRF at a population level. Previous studies have been limited to patients with a specific, primary diagnosis such chronic obstructive pulmonary disease (COPD), or receiving a particular intervention such as non‐invasive ventilation therapy.^1^, ^2^, ^3^, ^4^ These studies are prone to selection bias and imprecise estimates for the prevalence of each cause due to exclusion of potentially relevant participants, especially if participants have been selected based on survival of the acute episode. Few studies in recent years have reviewed causes of hypercapnia among hospitalized patients, without considering the source population.^5^, ^6^ Furthermore, there is increasing recognition that, in many instances, multiple causes can co‐exist.^1^ We propose that valid assessment of the prevalence of causes of HRF in the general population requires systematic evaluation of an unselected cohort of patients with hypercapnia whose inclusion is not biased with respect to primary diagnosis, interventions received or clinical outcome.

We sought to determine the prevalence of specific causes among patients with hypercapnia in the general population. We hypothesized that we would observe a diverse range of causes and that many patients would have ‘multifactorial’ HRF, with obstructive lung disease, sleep disordered breathing (SDB) and congestive cardiac failure (CCF) all potentially contributing to ventilatory failure. Our second objective was to assess the associations between specific causes and in‐hospital mortality. By providing these data, our goal was to provide context for clinicians involved in the care of such patients and to inform the development of appropriate management pathways focusing on key reversible causes for HRF in the community.

## METHODS

This cross‐sectional study was conducted at Liverpool Hospital, an 850‐bed, tertiary‐referral hospital with over 80,000 presentations to the Emergency Department each year.^7^ Based in Sydney, Australia, it is the major service provider for adult residents of the City of Liverpool, which in 2016 had an estimated population of 158,036 persons aged 15 years and over.^8^ State‐wide hospital admissions data show that 86% of residents from this region who are hospitalized for respiratory conditions attend Liverpool Hospital, the referral base for this study.^9^

The cohort comprised people presenting to Liverpool Hospital from 1st January 2013 to 31st December 2017 whose first arterial blood gas (ABG) sample taken within 24 h of presentation revealed PaCO_2_ >45 mm Hg and pH ≤7.45. To exclude probable venous specimens we excluded results where the blood gas SaO_2_ was at least 10% lower than pulse oximetry SpO_2_, if the latter was recorded within 2 hours of the former. We excluded confirmed or suspected nosocomial cases where the person had suffered an out‐of‐hospital cardiac arrest or traumatic injury, or if the specimen was collected during or shortly after a procedure requiring any sedatives or general anaesthesia (e.g., after an emergency surgical procedure).

**FIGURE 1:**
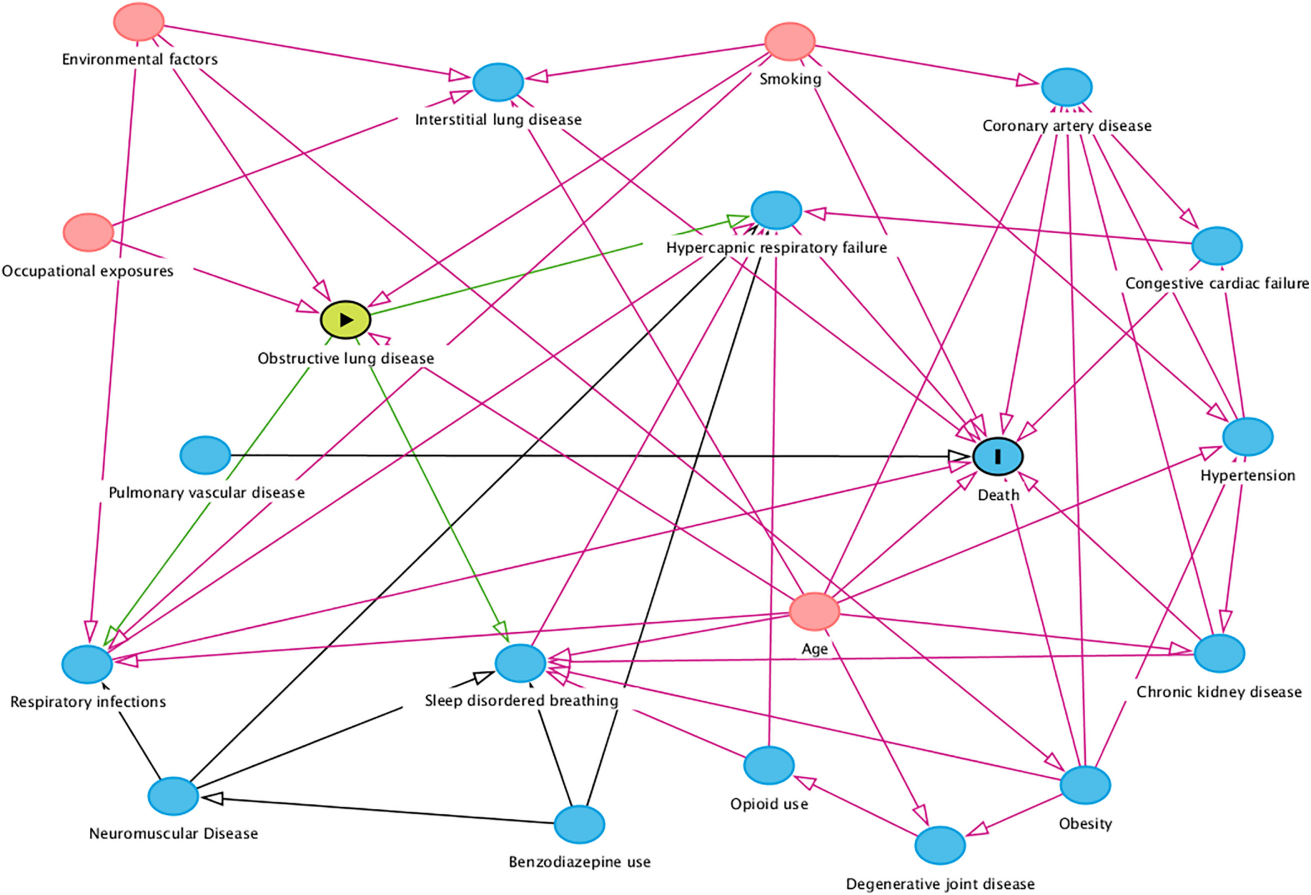
Causal diagram for hypercapnic respiratory failure and death. This directed acyclic graph explicitly states assumed causal relationships and allows identification of minimally sufficient adjustment sets for use in multivariable analyses. In this case, the graph has been constructed to estimate the effect of obstructive lung disease on death. Green arrows represent causal paths. Red arrows represent biasing paths. Blue‐shaded variables represent ancestors (causes) of the outcome (death). Red‐shaded variables represent ancestors (causes) of both the exposure (obstructive lung disease) and outcome (death).

For each case, we extracted demographic and clinical details, including the outcome of in‐hospital death, and data on potential contributing causes using electronically coded hospital records. Each cause was pre‐specified using a directed acyclic graph (DAG) developed by the authors showing both direct and indirect pathways for the development of HRF and subsequent death (Figure 1). We specified the following conditions as potentially directly antecedent to the development of HRF: obstructive lung disease (including COPD and asthma), SDB (obstructive sleep apnoea and obesity‐related hypoventilation syndromes), CCF, lower respiratory tract infection, neuromuscular disease (including chest wall disorders) and complications from opioid and/or benzodiazepine use. We used corresponding diagnosis codes to identify the presence or absence of these causes among study participants, as well as the degree of comorbidity based on the Charlson Comorbidity Index.^10^, ^11^ Where possible, we used existing algorithms validated for use in identifying medical conditions from clinical records (Table S1 in the Supporting Information).

We described the prevalence of causative conditions using frequencies and percentages. To estimate the associations between specific causes and the risk of death, we fitted separate logistic regression models with each hypothesised cause as the main effect (independent variable). The dependent variable was the binary outcome of death, compared with survival at hospital discharge. Using our DAG, we identified minimally sufficient adjustment sets (covariates) for inclusion in each model. If multiple adjustment sets were available, we avoided sets with latent (unmeasured) variables, such as environmental factors, and sets containing variables we expected to be poorly recorded, such as SDB. Arterial pH was used as a marker of HRF severity. Covariates for each regression model are provided in the Supporting Information (Figures S1–S6). Further discussion on causal pathway analysis and the use of DAGs can be found elsewhere.^12^, ^13^ We used the web‐based version of ‘daggity’^14^ to generate the DAG (Methods in the Supporting Information) and performed regression analyses in SAS (Version 9.4; SAS Institute Inc., Cary, NC).

## RESULTS

**FIGURE 2:**
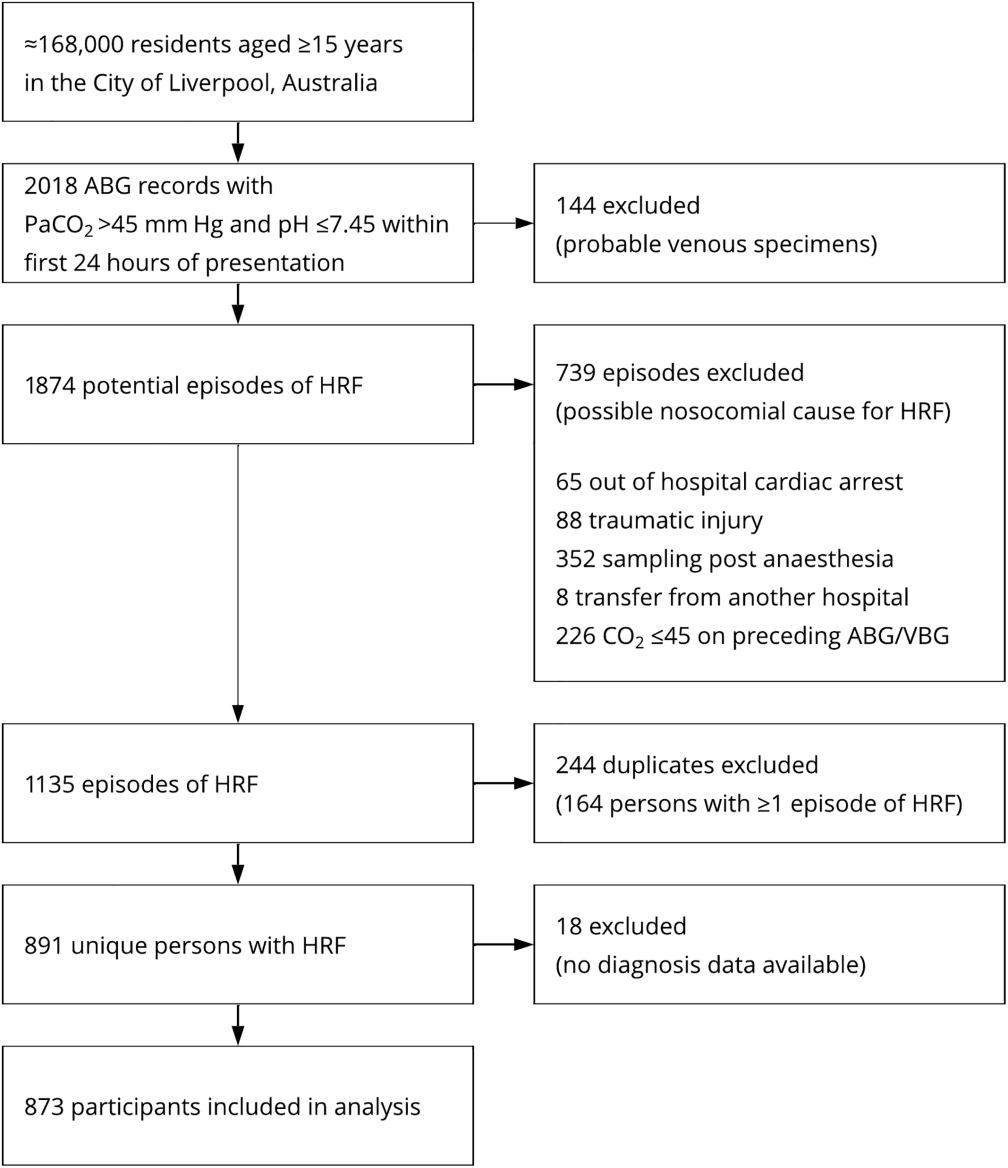
Flow chart demonstrating identification of cases of hypercapnic respiratory failure from 2013 to 2017 inclusive. ABG, arterial blood gas; HRF, hypercapnic respiratory failure; VBG, venous blood gas.

We identified 873 persons with HRF during the 5‐year study period (Figure 2). Patient characteristics are shown in Table 1.

**TABLE 1:** Characteristics of patients presenting with hypercapnic respiratory failure (*N* = 873)

Patient characteristic	*n* (%) unless otherwise specified
Age, mean (SD)	69 (17)
Sex	
Male	440 (50.4%)
Female	433 (49.6%)
History of smoking (tobacco) exposure	436 (49.9%)
Residence in aged care (nursing home)	72 (8.3%)
Charlson comorbidity index (CCI) score	
0	59 (6.8%)
1–2	116 (13.3%)
3–4	240 (27.5%)
5 or more	458 (52.4%)
Arterial blood gas values, mean (SD)	
pH	7.31 (0.09)
pO_2_	99 (65) mm Hg
pCO_2_	58 (14) mm Hg
Bicarbonate	29 (5.3) mmol/L
Acidosis (pH <7.35)	483 (55.3%)

At least one directly antecedent cause for HRF based on our model was identified in 724 (83%) of study participants (Table 2). The most frequently recorded cause was obstructive lung disease, observed in 389 (44.6%) cases. CCF was the most prevalent non‐respiratory diagnosis, potentially contributing to HRF in 278 (31.8%) cases. Adverse effects related to opioids and benzodiazepines were recorded in a minority of cases (6.5% and 3.0%, respectively), predominantly in younger age groups. SDB was recorded in 52 (6.0%) and neuromuscular disease in 15 (1.7%) cases. With respect to indirect causes for HRF, we identified a history of tobacco exposure in 436 (49.9%) cases but documented obesity in 47 (5.4%) cases only.

**TABLE 2:** Frequency and case fatality rate by antecedent cause among patients presenting with hypercapnic respiratory failure (*N* = 873)

Cause	Frequency *n* (%)	Death in hospital *n* (case fatality rate)
Obstructive lung disease	389 (44.6%)	36 (9.3%)
Lower respiratory tract infection	291 (33.3%)	53 (18.2%)
Congestive cardiac failure	278 (31.8%)	36 (13.0%)
Opioid use	57 (6.5%)	2 (3.5%)
Sleep disordered breathing	52 (6.0%)	1 (1.9%)
Benzodiazepine use	26 (3.0%)	0 (0%)
Neuromuscular disease	15 (1.7%)	6 (40.0%)

**FIGURE 3:**
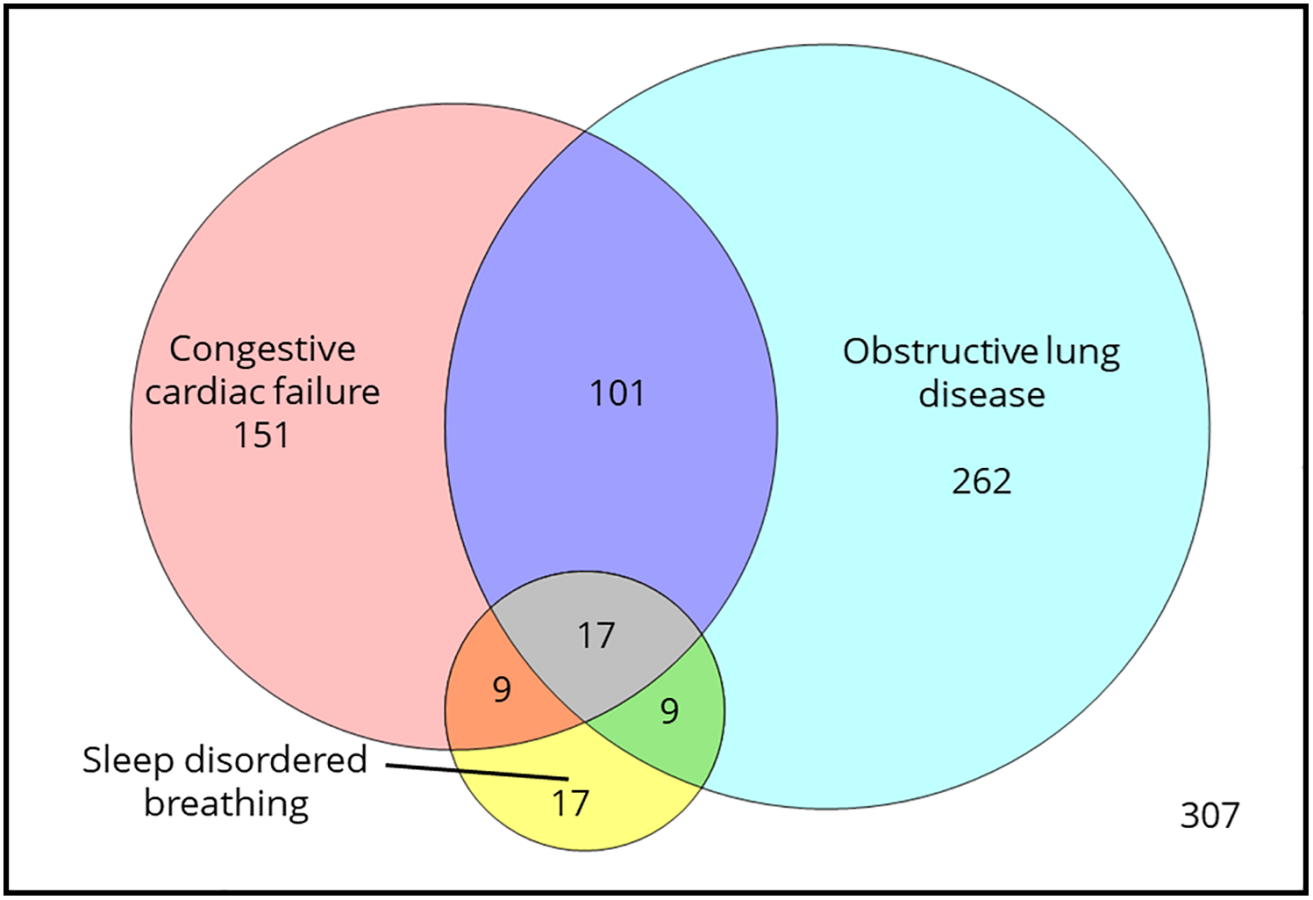
Prevalence of obstructive lung disease, congestive cardiac failure and sleep disordered breathing, either alone or in combination, among patients with hypercapnic respiratory failure (*N* = 873).

Among patients with obstructive lung disease, 118 (30%) had concomitant CCF and 26 (6.7%) had concomitant SDB. Conversely, among those with CCF, 118 (42%) had concomitant obstructive lung disease and 26 (9.4%) had SDB recorded. Only 17 (2.0%) of the entire cohort had all three of these conditions recorded concurrently (Figure 3).

The in‐hospital mortality rate was 12.8%. Most (94%) deaths occurred in patients aged 55 years and over. There was substantial variation in the case fatality rate among the various causes (Table 2).

The risks of death according to antecedent cause are presented in Table 3. After adjustment, the presence of obstructive lung disease or CCF was associated with a reduced risk of death, compared to those without these diagnoses. SDB also appeared to be associated with lower odds of death, though there was imprecision in the estimate (*p* = 0.06). In contrast, the presence of lower respiratory tract infection increased the risk of death. Neuromuscular disease conferred the highest risk of death among the causes assessed in this study.

**TABLE 3:** Associations between causes and in‐hospital death among patients with hypercapnic respiratory failure

	Risk of death if cause is present (95% CI)			
Cause	Unadjusted odds ratio	*p* value	Adjusted odds ratio	*p* value
Obstructive lung disease	0.55 (0.36–0.83)	<0.01	0.59 (0.36–0.98)	0.04
Lower respiratory tract infection	1.97 (1.32–2.95)	<0.01	1.68 (1.09–2.59)	0.02
Congestive cardiac failure	1.02 (0.66–1.55)	0.94	0.55 (0.34–0.90)	0.02
Opioid use	0.23 (0.06–0.97)	0.05	0.45 (0.10–1.94)	0.28
Sleep disordered breathing	0.13 (0.02–0.92)	0.04	0.14 (0.02–1.11)	0.06
Neuromuscular disease	4.73 (1.65–13.6)	<0.01	8.02 (2.56–25.2)	<0.01

## DISCUSSION

Our study demonstrates that HRF is frequently associated with obstructive lung disease and CCF, either alone or in combination, as well as a range of lesser common causes. In contrast to previous work, we found that few patients had ‘multifactorial’ HRF with at least three antecedent causes. Importantly, we found that the underlying aetiology for HRF had a significant impact on in‐hospital mortality. This is the first study to examine the causes of HRF at a population level, providing valuable information to assist clinicians caring for such patients and baseline data for future studies on HRF.

We found that obstructive lung disease was the most frequently recorded chronic condition antecedent to HRF. This finding is consistent with studies of patients with HRF referred for ventilatory support, which typically find COPD as the most common condition.^1^, ^2^, ^3^ In a recent study of 78 survivors of acute HRF, 52 (67%) had airflow limitation. However, only 19 (24%) had known COPD based on spirometry.^1^ The reliance on spirometry to confirm COPD may be overstated in the acute setting; in another series of patients with respiratory acidaemia, nearly 80% of patients with a clinical diagnosis of COPD were subsequently confirmed to have airflow obstruction.^15^ Our findings underscore the importance of obstructive lung disease as an aetiological factor for HRF and provide support for its assumed presence in most patients with ventilatory failure. Future research should focus on assessing the predictive value of clinical features, either alone or in combination as part of clinician gestalt, which may indicate responsiveness to treatments such as bronchodilators and corticosteroids among patients presenting with HRF.

A substantial proportion of our study population had non‐respiratory causes, the most common being CCF. Although not typically associated with hypercapnia, systolic heart failure may lead to ventilatory failure by a number of mechanisms including dynamic airflow obstruction which resolves following the acute episode.^16^ Up to 44% of patients requiring ventilatory support for HRF have cardiac dysfunction and/or cardiogenic pulmonary oedema.^1^, ^3^, ^17^ and hypercapnia occurs in up to 58% of patients with acute heart failure.^18^, ^19^ Given that patients with COPD are at increased risk of heart failure, the presence of which is associated with worse prognosis.^20^ a bipartisan approach is required to develop investigation pathways for patients with HRF that take into account both of these common conditions.

Unlike previous studies, we found that few patients had ‘multifactorial’ HRF. The discrepancy is probably attributable to the very low prevalence of SDB observed in our cohort. Prior studies have found that severe SDB is present in up to 83% of HRF survivors.^1^, ^21^ Our value of 6.0% is most likely an underestimate due to incomplete recording of the diagnosis, a well‐recognized issue with detecting sleep disorders using medical records.^22^ Furthermore, SDB may be underrecognized in patients with HRF,^23^ at least in part due to the specialized nature of diagnostic testing which may not be equally accessible to all members of the community. Further work is required, ideally using long‐term cohort data, to establish antecedent causes prior to the development of hypercapnia to confirm the true prevalence of multifactorial HRF. Multiple sufficient cause sets may exist, with some patients having one condition at an advanced stage (particularly COPD) and others having multiple co‐existent diseases of lesser severity. Understanding the role that specific diagnoses play will allow clinicians to develop more effective treatment pathways for patients with undifferentiated HRF.

Our study found that the aetiology of HRF had a significant impact on in‐hospital death. To our knowledge, this is the first study to demonstrate that the presence of COPD and heart failure are associated with a lower risk of death, whereas respiratory tract infections and neuromuscular disease are associated with increased risk of death. Although hypercapnia is associated with a worse prognosis among patients with a specific diagnosis such as COPD,^24^ there are scarce data on the impact of these diagnoses on those with undifferentiated HRF or HRF due to multiple causes. A study of 202 patients with HRF found that although the degree of comorbidity could predict survival after hospital discharge, there were no differences in comorbidities among survivors and those who died during admission.^25^ A potential explanation for our results is the increased availability and use of non‐invasive ventilation therapy for patients with HRF. Systematic reviews have confirmed the mortality benefit conferred by non‐invasive ventilation therapy among patients with COPD and acute cardiogenic pulmonary oedema.^26^, ^27^ Another explanation is that the observed risk reduction is relative to the risk of death in all patients with HRF as a whole; COPD and heart failure are less lethal compared with neuromuscular disease. Appreciating the impact of comorbidities among patients with HRF may allow for a greater degree of informed decision making particularly with respect to goals of care and avoiding potentially futile medical interventions. Our results suggest that patients with HRF should not necessarily have treatment withheld for fear of worse outcomes based solely on the presence of COPD or heart failure. Future studies performed on patients who survive an episode of HRF should also consider the potential selection bias resulting from overrepresentation of patients with chronic cardiopulmonary disease and underrepresentation of neuromuscular conditions.

A key strength of our study is the focus on an unselected cohort identified based on ABG measurements. Case identification is an inherent difficulty for any study of patients with HRF, as there is no specific diagnosis code for this condition.^28^ We have chosen to consider both acute and chronic forms of HRF together as the duration of ‘chronic’ HRF can be variable, and there may be significant overlap in causes. Our method has good face validity, and by including patients with all causes we provide unique insights into contributing factors at a population level, appreciating that most people with respiratory illness requiring hospitalization in this population will be captured in this study. Furthermore, our study is unique in its use of theorized causal pathways to inform regression models for HRF and death. The model is necessarily complex and may require adjustment based on future research. However, it provides a foundation for studies to test the relevance of hypothesised causal pathways (including multifactorial causal sets) and the effects of interventions to mitigate adverse outcomes associated with HRF.

Our study has some limitations. Being a retrospective study, we have relied on diagnosis codes for the detection of causes, and hence have no data on disease specifics, duration or severity. Under‐diagnosis is likely to be present, especially for SDB as discussed above. However, in many instances, direct measurement of causes requires patients to survive the acute episode and/or achieve a degree of clinical stability whilst hospitalized. The mortality rate for our cohort is higher than that observed in previous smaller studies^25^, ^29^ and highlights the potential for survivorship bias when causes are directly measured. Next, it is possible we may have missed HRF cases in whom an ABG was not performed. We suspect the number of cases missed in hospital to be low as we typically obtain an ABG for all patients in whom there is a clinical suspicion for HRF, and do not use venous blood gas or other measurements to confirm hypercapnia. The relative difficulty of obtaining arterial blood gases to confirm hypercapnia is one of the primary reasons for the paucity of data on this topic, and we acknowledge that our study will have missed community‐dwelling patients with hypercapnia who do not attend hospital. Finally, our results may have limited generalizability, depending on the comparability of other contexts to our source population.

In summary, we found HRF to be associated with a range of causes which appeared to have independent, significant effects on hospital survival. Clinicians making management decisions for patients with HRF should incorporate information on underlying conditions when making management decisions. This is the first report of HRF as a single entity assessed at a population level, and provides context to previous work on HRF survivors which may be subject to selection bias. Further work is required particularly with respect to understanding sufficient cause sets for HRF to guide interventions directed at prevention and management.

## AUTHOR CONTRIBUTION

**Yewon Chung:** Conceptualization (equal); data curation (equal); formal analysis (equal); funding acquisition (equal); investigation (equal); methodology (equal); project administration (equal); writing – original draft (equal); writing – review and editing (equal). **Frances L. Garden:** Data curation (equal); formal analysis (equal); funding acquisition (equal); investigation (equal); methodology (equal); resources (equal); software (equal); supervision (equal); writing – review and editing (equal). **Guy Marks:** Conceptualization (equal); formal analysis (equal); funding acquisition (equal); investigation (equal); methodology (equal); software (equal); supervision (equal); writing – original draft (equal); writing – review and editing (equal). **Hima Vedam:** Conceptualization (equal); data curation (equal); funding acquisition (equal); investigation (equal); methodology (equal); project administration (equal); resources (equal); supervision (equal); writing – original draft (equal); writing – review and editing (equal).

## CONFLICT OF INTEREST

None declared.

## HUMAN ETHICS APPROVAL DECLARATION

This study was approved by the South Western Sydney Local Health District Human Research Ethics Committee (Reference LNR/17/LPOOL/282). A waiver of consent was granted as this was a non‐interventional study and the benefits justified any risks of harm associated with not seeking consent.

## Supporting information


**Table S1.** Diagnosis codes from the International Classification of Diseases and Related Health Problems, Tenth Revision, Australian Modification (ICD‐10‐AM) used for data abstraction.
**Figure S1.** Causal model for the effective of obstructive lung disease on death. Green arrows represent causal paths. Red arrows represent biasing paths. Blue‐shaded variables represent ancestors (causes) of the outcome (death). Red‐shaded variables represent ancestors (causes) of both the exposure (obstructive lung disease) and outcome (death). Based on this graph, the following covariates were included in the final regression model: Age, pH, chronic kidney disease, congestive cardiac failure, interstitial lung disease, obesity, respiratory infection and smoking.
**Figure S2.** Causal model for the effect of lower respiratory tract infection on death. Green arrows represent causal paths. Red arrows represent biasing paths. Blue‐shaded variables represent ancestors (causes) of the outcome (death). Red‐shaded variables represent ancestors (causes) of both the exposure (lower respiratory tract infection) and outcome (death). Based on this graph, the following covariates were included in the final regression model: Age, pH, benzodiazepine use, congestive cardiac failure, interstitial lung disease, neuromuscular disease, obesity, obstructive lung disease, opioid use, sleep disordered breathing, and smoking.
**Figure S3.** Causal model for the effect of congestive cardiac failure on death. Green arrows represent causal paths. Red arrows represent biasing paths. Blue‐shaded variables represent ancestors (causes) of the outcome (death). Red‐shaded variables represent ancestors (causes) of both the exposure (congestive cardiac failure) and outcome (death). Based on this graph, the following covariates were included in the final regression model: Age, pH, chronic kidney disease, coronary artery disease, interstitial lung disease, obesity, respiratory infection, and smoking.
**Figure S4.** Causal model for the effect of opioid use on death. Green arrows represent causal paths. Red arrows represent biasing paths. Blue‐shaded variables represent ancestors (causes) of the outcome (death). Red‐shaded variables represent ancestors (causes) of both the exposure (opioid use) and outcome (death). Based on this graph, the following covariates were included in the final regression model: Age, pH, chronic kidney disease, congestive cardiac failure, neuromuscular disease, obesity, obstructive airways disease, and respiratory infection.
**Figure S5.** Causal model for the effect of sleep disordered breathing on death. Green arrows represent causal paths. Red arrows represent biasing paths. Blue‐shaded variables represent ancestors (causes) of the outcome (death). Red‐shaded variables represent ancestors (causes) of both the exposure (sleep disordered breathing) and outcome (death). Based on this graph, the following covariates were included in the final regression model: Age, pH, chronic kidney disease, congestive cardiac failure, neuromuscular disease, obesity, obstructive airways disease, and respiratory infection.
**Figure S6.** Causal model for the effect of neuromuscular disease on death. Green arrows represent causal paths. Red arrows represent biasing paths. Blue‐shaded variables represent ancestors (causes) of the outcome (death). Red‐shaded variables represent ancestors (causes) of both the exposure (neuromuscular disease) and outcome (death). Based on this graph, the following covariates were included in the final regression model: Age, pH, chronic kidney disease, congestive cardiac failure, interstitial lung disease, obesity, respiratory infection, and sleep disordered breathing.

**Visual Abstract** Causes of hypercapnic respiratory failure and associated in‐hospital mortality

## Data Availability

The data that support the findings of this study are available on request from the corresponding author. The data are not publicly available due to privacy or ethical restrictions.
