# Supplementary material for: Causes of hypercapnic respiratory failure and associated in‐hospital mortality
Source: Respirology. 2022 Oct 9;28(2):176–82. doi: 10.1111/resp.14388 (PMC10092076; doi:10.1111/resp.14388)
Supplement: Supplementary file 1 — Table S1. Diagnosis codes from the International Classification of Diseases and Related Health Problems, Tenth Revision, Australian Modification (ICD‐10‐AM) used for data abstraction. Figure S1. Causal model for the effective of obstructive lung disease on death. Green arrows represent causal paths. Red arrows represent biasing paths. Blue‐shaded variables represent ancestors (causes) of the outcome (death). Red‐shaded variables represent ancestors (causes) of both the exposure (obstructive lung disease) and outcome (death). Based on this graph, the following covariates were included in the final regression model: Age, pH, chronic kidney disease, congestive cardiac failure, interstitial lung disease, obesity, respiratory infection and smoking. Figure S2. Causal model for the effect of lower respiratory tract infection on death. Green arrows represent causal paths. Red arrows represent biasing paths. Blue‐shaded variables represent ancestors (causes) of the outcome (death). Red‐shaded variables represent ancestors (causes) of both the exposure (lower respiratory tract infection) and outcome (death). Based on this graph, the following covariates were included in the final regression model: Age, pH, benzodiazepine use, congestive cardiac failure, interstitial lung disease, neuromuscular disease, obesity, obstructive lung disease, opioid use, sleep disordered breathing, and smoking. Figure S3. Causal model for the effect of congestive cardiac failure on death. Green arrows represent causal paths. Red arrows represent biasing paths. Blue‐shaded variables represent ancestors (causes) of the outcome (death). Red‐shaded variables represent ancestors (causes) of both the exposure (congestive cardiac failure) and outcome (death). Based on this graph, the following covariates were included in the final regression model: Age, pH, chronic kidney disease, coronary artery disease, interstitial lung disease, obesity, respiratory infection, and smoking. Figure S4. Causal model for t [file RESP-28-176-s001.docx]

**Supporting Information**

Causes of hypercapnic respiratory failure and associated in-hospital mortality

Yewon Chung, Frances L. Garden, Guy B. Marks, Hima Vedam.

**Contents**

| **Supplementary Table S1.** Diagnosis codes from the International Classification of Diseases and Related Health Problems, Tenth Revision, Australian Modification (ICD-10-AM) used for data abstraction | **Page 2** |
| --- | --- |
| **Supplementary Methods**. Proposed causal model for hypercapnic respiratory failure and death | **Page 4** |
| **Supplementary Figure S1.** Causal model for the effective of obstructive lung disease on death. | **Page 6** |
| **Supplementary Figure S2.** Causal model for the effect of lower respiratory tract infection on death. | **Page 7** |
| **Supplementary Figure S3.** Causal model for the effect of congestive cardiac failure on death. | **Page 8** |
| **Supplementary Figure S4.** Causal model for the effect of opioid use on death. | **Page 9** |
| **Supplementary Figure S5.** Causal model for the effect of sleep disordered breathing on death. | **Page 10** |
| **Supplementary Figure S6.** Causal model for the effect of neuromuscular disease on death. | **Page 11** |
| **References** | **Page 12** |

**Table S1.** Diagnosis codes from the International Classification of Diseases and Related Health Problems, Tenth Revision, Australian Modification (ICD-10-AM) used for data abstraction.

| **Condition associated with hypercapnic respiratory failure** | **ICD Codes used for data extraction** |
| --- | --- |
| Benzodiazepine use | T42.4,Y47.1 |
| Chronic kidney disease | I12.0, I12.9, I13.0, I13.1, I13.2, I13.9, N18.1, N18.2, N18.3, N18.4, N18.5, N18.9, Z99.2 |
| Congestive cardiac failure | I09.0, I11.0, I13.0, I13.2, I25.5, I42.0, I42.1, I42.2, I42.3, I42.4, I42.5, I42.6, I42.7, I42.8, I42.9, I43.0, I43.1, I43.2, I43.8, I50.0, I50.1, I50.9 |
| Coronary artery disease | I21.0, I21.1, I21.2, I21.3, I21.4, I21.9, I22.0, I22.1, I22.8, I22.9, I24.0, I24.8, I24.9, I25.0, I25.1, I25.10, I25.11, I25.12, I25.13, I25.2, I25.4, I25.5, I25.6, I25.8, I25.9 |
| Hypertension | I10, I11.0, I11.9, I12.0, I12.9, I13.0, I13.1, I13.2, I13.9, I15.0, I15.1, I15.2, I15.8, I15.9 |
| Interstitial lung disease | J60, J61, J62, J62.0, J62.8, J63, J63.0, J63.1, J63.2, J63.3, J63.4, J63.5, J63.8, J64, J65, J70.1, J70.3, J70.4, J84.1, J84.9, J92.0, J99.1, J99.8 |
| Neuromuscular disease | G12.0, G12.1, G12.2, G12.8, G12.9, G61, G61.0, G61.8, G61.9, G70.0, G70.1, G70.2, G70.8, G70.9, G71.0, G71.1, G71.2, G71.3, G71.8, G71.9, G82.3, G82.30, G82.31, G82.32, G82.33, G82.34, G82.35, G82.36, G82.4, G82.40, G82.41, G82.42, G82.43, G82.44, G82.45, G82.46, G82.5, G82.51, G82.52, G82.53, G82.54, 82.55, G82.56, J98.6 |
| Obesity | E66, E66.1, E66.10, E66.11, E66.12, E66.13, E66.2, E66.20, E66.21, E66.22, E66.23, E66.8, E66.9, E66.90, E66.91, E66.92, E66.93 |
| Obstructive lung disease | J43.1, J43.2, J43.8, J43.9, J44.0, J44.1, J44.8, J44.9, J45.0, J45.1,  J45.8, J45.9, J46, J47 |
| Opioid use | F11.0, F11.1, F11.2, F11.3, F11.4, F11.5, F11.6, F11.7, F11.8,  F11.9, T40.0, T40.1, T40.2, T40.3, T40.4, T40.6, Y45.0 |
| Lower respiratory tract infection | J09, J10, J10.0, J10.1, J10.8, J11.0, J11.1, J11.8, J12.0, J12.1, J12.2, J12.3, J12.8, J12.9, J13, J14, J15.0, J15.1, J15.2, J15.3, J15.4, J15.5, J15.6, J15.7, J15.8, J15.9, J16.0, J16.8, J17.0, J17.1, J17.2, J17.3, J17.8, J18.0, J18.1, J18.2, J18.8, J18.9, J20.0, J20.1, J20.2, J20.3, J20.4, J20.5, J20.6, J20.7, J20.8, J20.9, J21.0, J21.1, J21.8, J21.9, J22, J69.0, A15.0, A15.1, A15.2, A15.3, A15.5, A15.7, A15.8, A15.9, A16.0, A16.1, A16.2, A16.4, A16.7, A16.9, A16.9, A21.2, A22.1, A37.0, A37.1, A37.8, A37.9, A42.0, A43.0, A48.1, B25.0, B37.1, B38.0, B38.1, B38.2, B39.0, B39.1, B39.2, B40.0, B40.1, B40.2, B41.0, B42.0, B44.0, B44.1, B45.0, B46.0, B48.5 |
| Sleep disordered breathing | G47.30, G47.33, E66.2, E66.20, E66.21, E66.22, E66.23 |
| Smoking | F17.0, F17.1, F17.2, F17.3, F17.4, F17.5, F17.6, F17.7, F17.8, F17.9, T65.2, Z58.7, Z71.6, Z72.0, Z86.43 |

**Appendix S1 –** Methods: Proposed model for hypercapnic respiratory failure and death.

The following code may be entered into *Daggity*. We used the web-based version, accessed at « <http://www.dagitty.net/dags.html> »

dag {bb="-4.292,-3.673,4.369,4.361"

"Benzodiazepine use" [pos="-0.441,3.364"]

"Chronic kidney disease" [pos="3.298,1.936"]

"Congestive cardiac failure" [pos="3.286,-1.486"]

"Coronary artery disease" [pos="2.405,-2.810"]

"Degenerative joint disease" [pos="1.668,3.543"]

"Environmental factors" [pos="-3.020,-3.361"]

"Hypercapnic respiratory failure" [pos="0.708,-1.784"]

"Interstitial lung disease" [pos="-0.921,-2.855"]

"Neuromuscular Disease" [pos="-2.819,3.126"]

"Obstructive lung disease" [exposure,pos="-1.814,-0.861"]

"Occupational exposures" [pos="-3.320,-1.590"]

"Opioid use" [pos="0.664,2.873"]

"Pulmonary vascular disease" [pos="-2.629,0.270"]

"Respiratory infections" [pos="-3.321,2.025"]

"Sleep disordered breathing" [pos="-0.787,2.010"]

Age [pos="0.931,1.579"]

Death [outcome,pos="2.003,0.284"]

Hypertension [pos="3.465,0.121"]

Obesity [pos="2.516,3.037"]

Smoking [pos="0.786,-3.197"]

"Benzodiazepine use" -> "Hypercapnic respiratory failure"

"Benzodiazepine use" -> "Neuromuscular Disease"

"Benzodiazepine use" -> "Sleep disordered breathing"

"Chronic kidney disease" -> "Coronary artery disease"

"Chronic kidney disease" -> "Sleep disordered breathing"

"Chronic kidney disease" -> Death

"Congestive cardiac failure" -> "Hypercapnic respiratory failure"

"Congestive cardiac failure" -> Death

"Coronary artery disease" -> "Congestive cardiac failure"

"Coronary artery disease" -> Death

"Degenerative joint disease" -> "Opioid use"

"Environmental factors" -> "Interstitial lung disease"

"Environmental factors" -> "Obstructive lung disease"

"Environmental factors" -> "Respiratory infections"

"Environmental factors" -> Obesity

"Hypercapnic respiratory failure" -> Death

"Interstitial lung disease" -> Death

"Neuromuscular Disease" -> "Hypercapnic respiratory failure"

"Neuromuscular Disease" -> "Respiratory infections"

"Neuromuscular Disease" -> "Sleep disordered breathing"

"Obstructive lung disease" -> "Hypercapnic respiratory failure"

"Obstructive lung disease" -> "Respiratory infections"

"Obstructive lung disease" -> "Sleep disordered breathing"

"Occupational exposures" -> "Interstitial lung disease"

"Occupational exposures" -> "Obstructive lung disease"

"Opioid use" -> "Hypercapnic respiratory failure"

"Opioid use" -> "Sleep disordered breathing"

"Pulmonary vascular disease" -> Death

"Respiratory infections" -> "Hypercapnic respiratory failure"

"Respiratory infections" -> Death

"Sleep disordered breathing" -> "Hypercapnic respiratory failure"

Age -> "Chronic kidney disease"

Age -> "Coronary artery disease"

Age -> "Degenerative joint disease"

Age -> "Interstitial lung disease"

Age -> "Obstructive lung disease"

Age -> "Respiratory infections"

Age -> "Sleep disordered breathing"

Age -> Death

Age -> Hypertension

Hypertension -> "Chronic kidney disease"

Hypertension -> "Congestive cardiac failure"

Hypertension -> "Coronary artery disease"

Obesity -> "Coronary artery disease"

Obesity -> "Degenerative joint disease"

Obesity -> "Sleep disordered breathing"

Obesity -> Death

Obesity -> Hypertension

Smoking -> "Coronary artery disease"

Smoking -> "Interstitial lung disease"

Smoking -> "Obstructive lung disease"

Smoking -> "Respiratory infections"

Smoking -> Death

Smoking -> Hypertension}

**Figure S1.** Causal model for the effective of obstructive lung disease on death. Green arrows represent causal paths. Red arrows represent biasing paths. Blue-shaded variables represent ancestors (causes) of the outcome (death). Red-shaded variables represent ancestors (causes) of both the exposure (obstructive lung disease) and outcome (death). Based on this graph, the following covariates were included in the final regression model: Age, pH, chronic kidney disease, congestive cardiac failure, interstitial lung disease, obesity, respiratory infection, smoking.


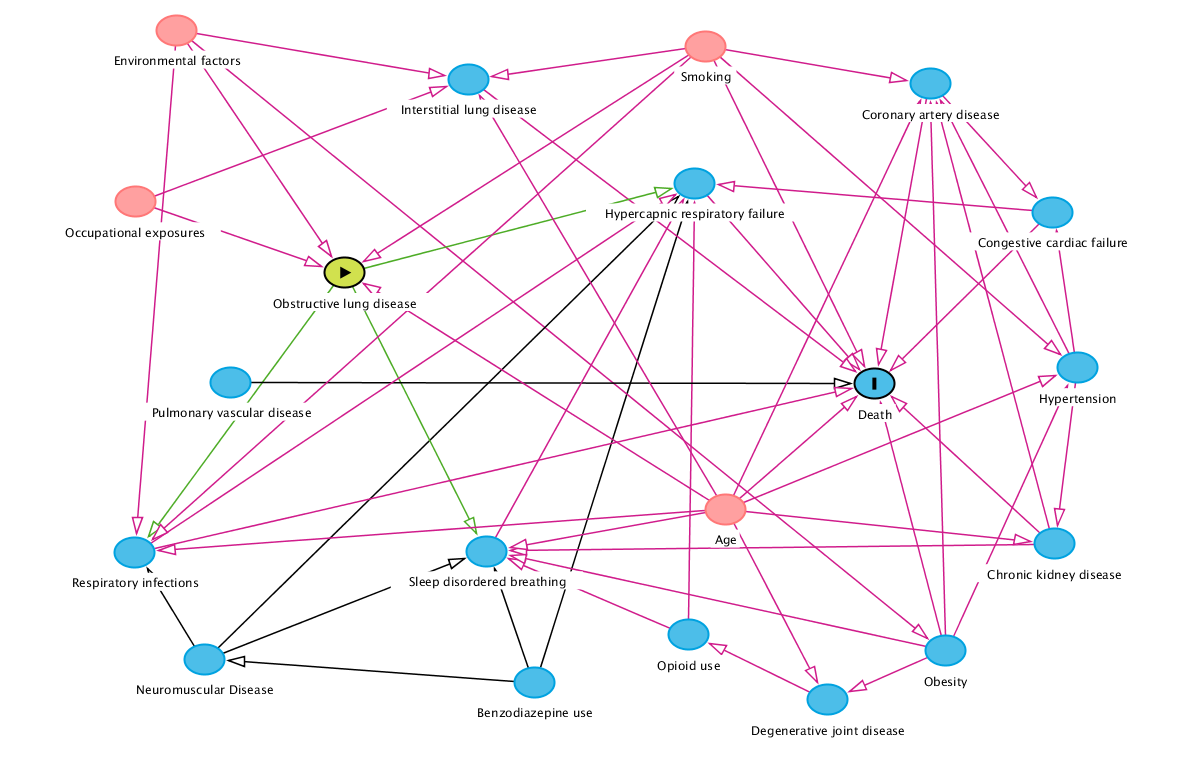


**Figure S2.** Causal model for the effect of lower respiratory tract infection on death. Green arrows represent causal paths. Red arrows represent biasing paths. Blue-shaded variables represent ancestors (causes) of the outcome (death). Red-shaded variables represent ancestors (causes) of both the exposure (lower respiratory tract infection) and outcome (death). Based on this graph, the following covariates were included in the final regression model: Age, pH, benzodiazepine use, congestive cardiac failure, interstitial lung disease, neuromuscular disease, obesity, obstructive lung disease, opioid use, sleep disordered breathing, smoking.


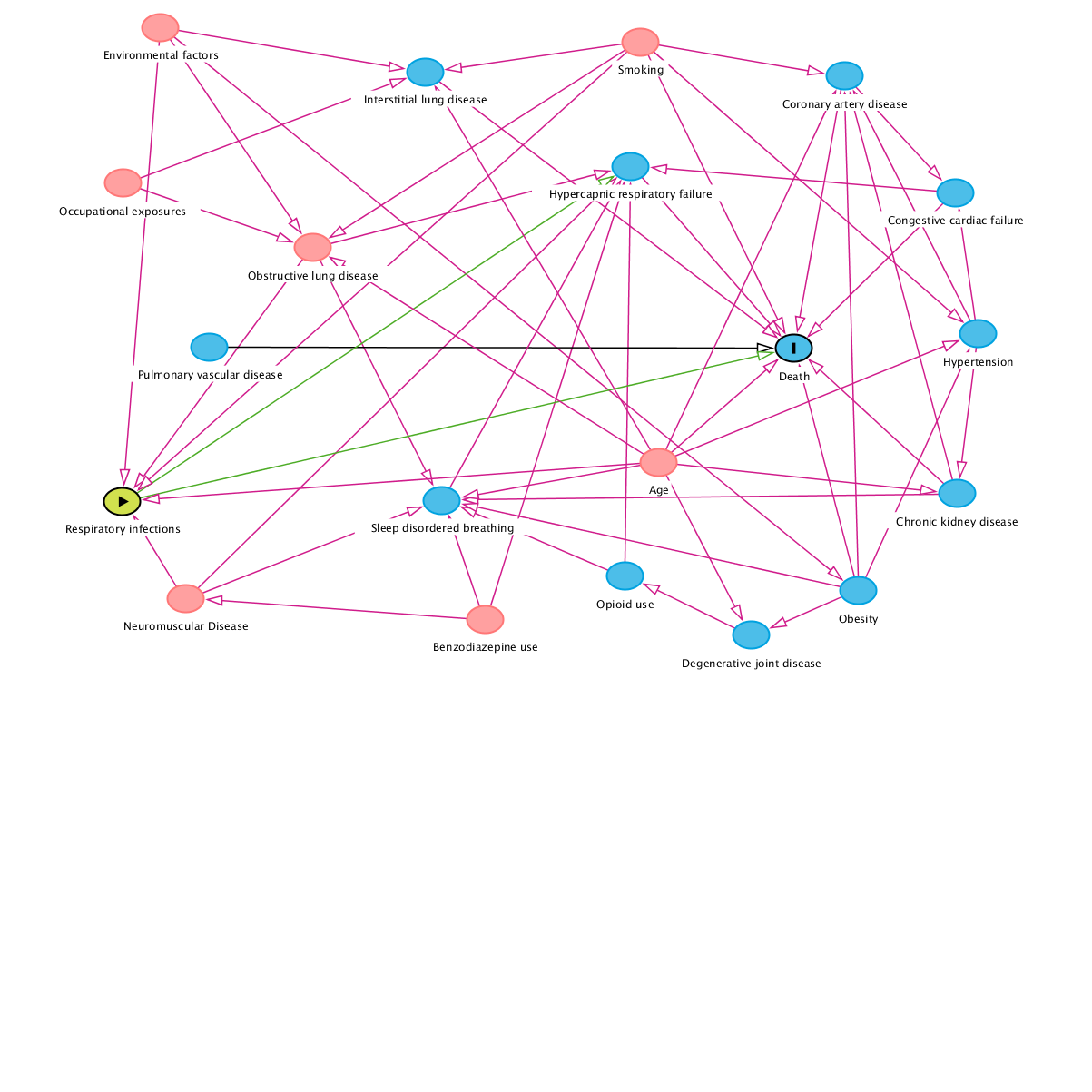


**Figure S3.** Causal model for the effect of congestive cardiac failure on death. Green arrows represent causal paths. Red arrows represent biasing paths. Blue-shaded variables represent ancestors (causes) of the outcome (death). Red-shaded variables represent ancestors (causes) of both the exposure (congestive cardiac failure) and outcome (death). Based on this graph, the following covariates were included in the final regression model: Age, pH, chronic kidney disease, coronary artery disease, interstitial lung disease, obesity, respiratory infection, smoking.


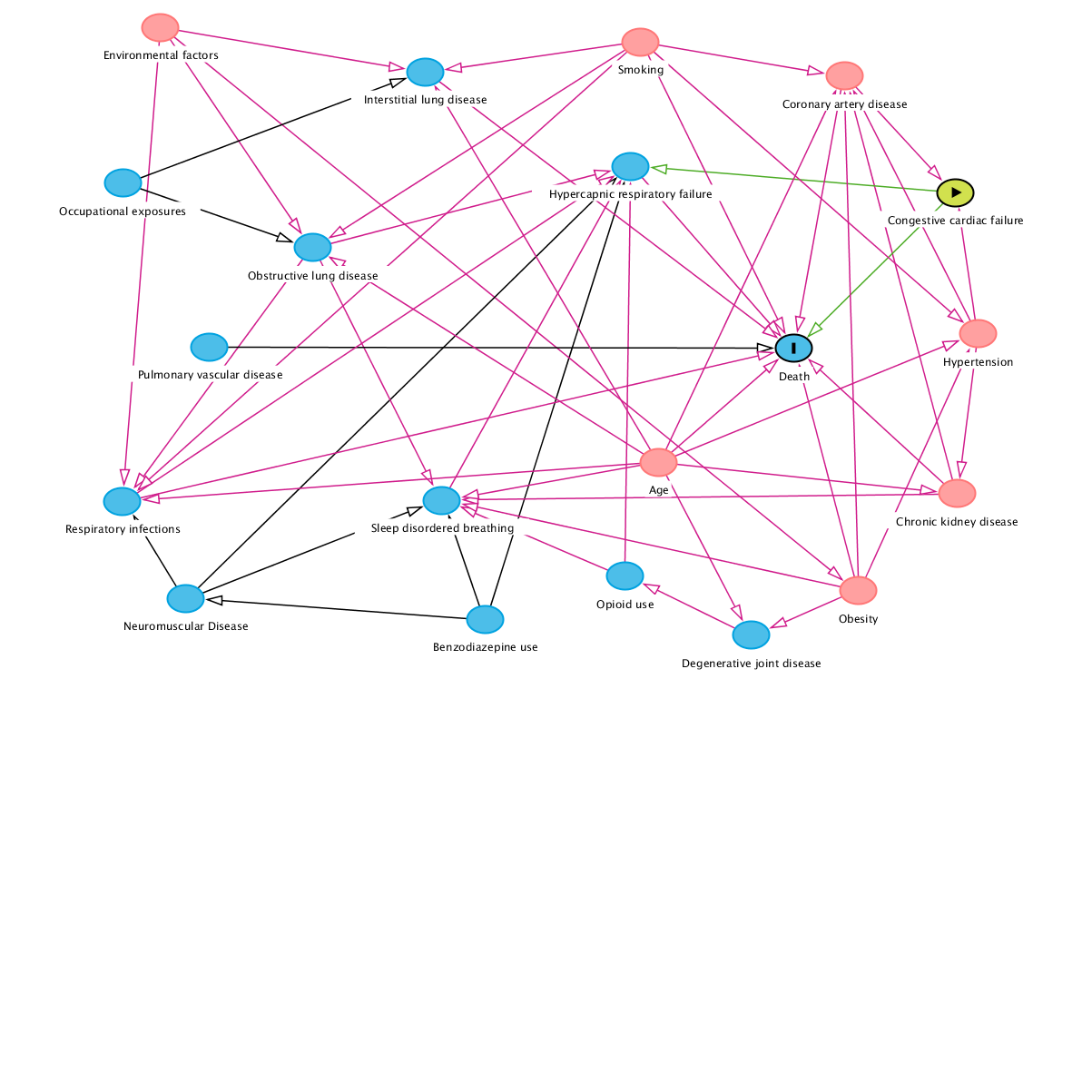


**Figure S4.** Causal model for the effect of opioid use on death. Green arrows represent causal paths. Red arrows represent biasing paths. Blue-shaded variables represent ancestors (causes) of the outcome (death). Red-shaded variables represent ancestors (causes) of both the exposure (opioid use) and outcome (death). Based on this graph, the following covariates were included in the final regression model: Age, pH, chronic kidney disease, congestive cardiac failure, neuromuscular disease, obesity, obstructive airways disease, respiratory infection.


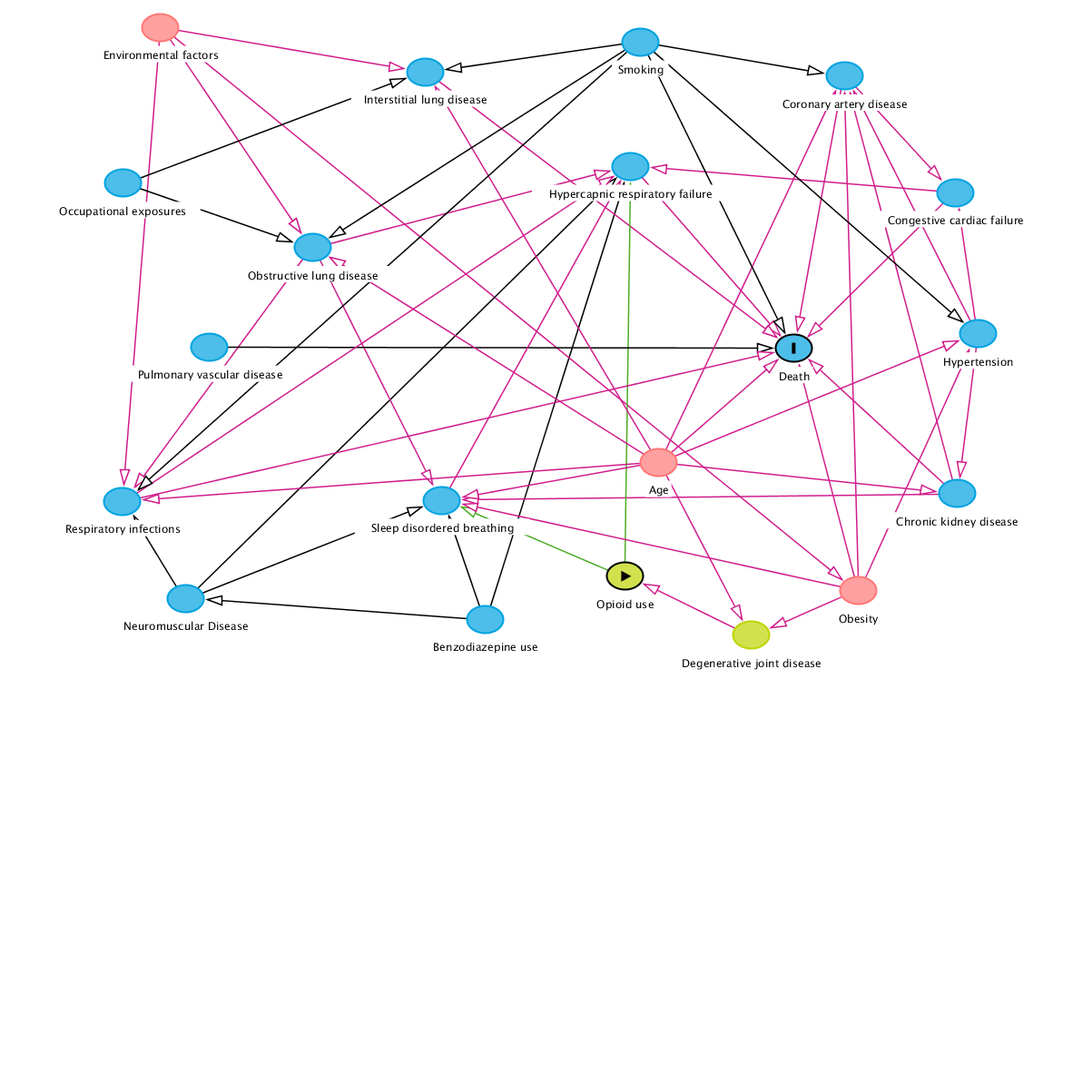


**Figure S5.** Causal model for the effect of sleep disordered breathing on death. Green arrows represent causal paths. Red arrows represent biasing paths. Blue-shaded variables represent ancestors (causes) of the outcome (death). Red-shaded variables represent ancestors (causes) of both the exposure (sleep disordered breathing) and outcome (death). Based on this graph, the following covariates were included in the final regression model: Age, pH, chronic kidney disease, congestive cardiac failure, neuromuscular disease, obesity, obstructive airways disease, respiratory infection.


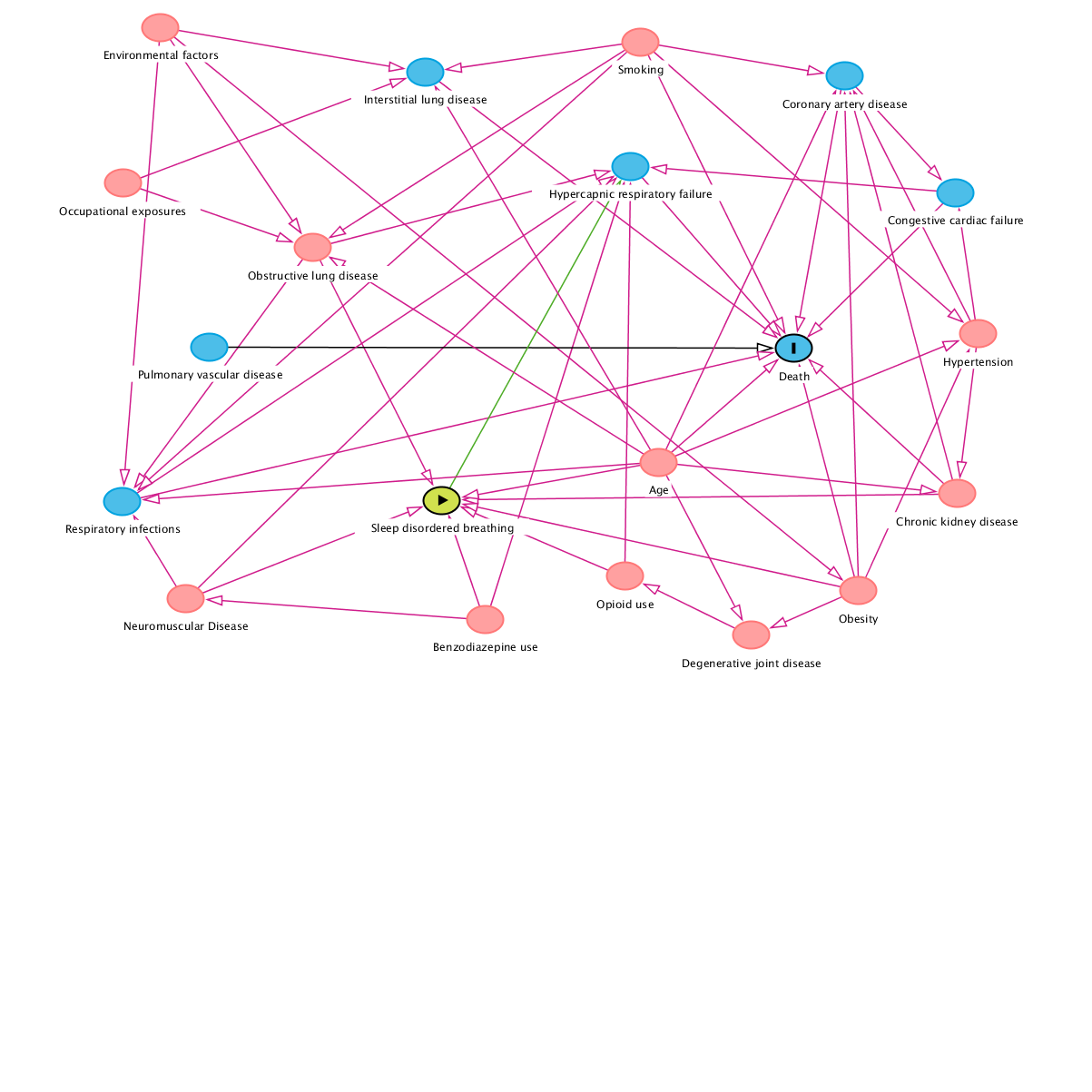


**Figure S6.** Causal model for the effect of neuromuscular disease on death. Green arrows represent causal paths. Red arrows represent biasing paths. Blue-shaded variables represent ancestors (causes) of the outcome (death). Red-shaded variables represent ancestors (causes) of both the exposure (neuromuscular disease) and outcome (death). Based on this graph, the following covariates were included in the final regression model: Age, pH, chronic kidney disease, congestive cardiac failure, interstitial lung disease, obesity, respiratory infection, sleep disordered breathing.


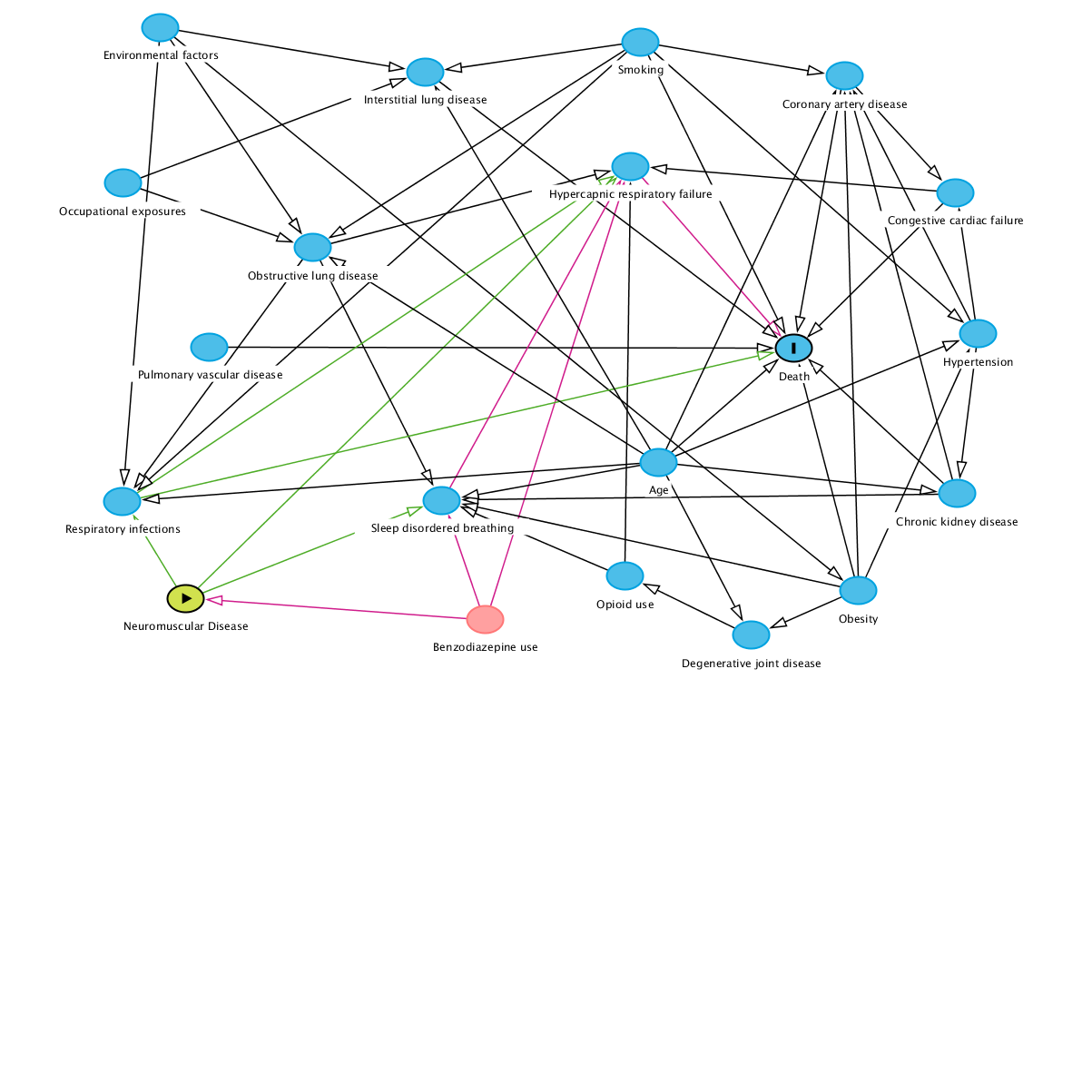


**References**

1. The Independent Hospital Pricing Authority. The International Statistical Classification of Diseases and Related Health Problems, Tenth Revision, Australian Modification (ICD-10-AM). Independent Hospital Pricing Authority, Darlinghurst, NSW.

2. Textor J, van der Zander B, Gilthorpe MS, Liśkiewicz M, Ellison GT. Robust causal inference using directed acyclic graphs: the R package ‘dagitty.’ *International Journal of Epidemiology* 2016;45:1887–1894.
