# Supplementary material for: Causes of hypercapnic respiratory failure and associated in‐hospital mortality
Source: Respirology. 2022 Oct 9;28(2):176–82. doi: 10.1111/resp.14388 (PMC10092076; doi:10.1111/resp.14388)
Supplement: Supplementary file 2 — Visual Abstract Causes of hypercapnic respiratory failure and associated in‐hospital mortality [file RESP-28-176-s002.pdf]

Causes of hypercapnic respiratory failure and associated in-hospital mortality

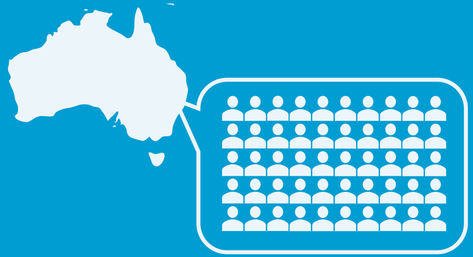

$PaCO_2 > 45$ mmHg
pH ≤ 7.45

Mortality
12.8%

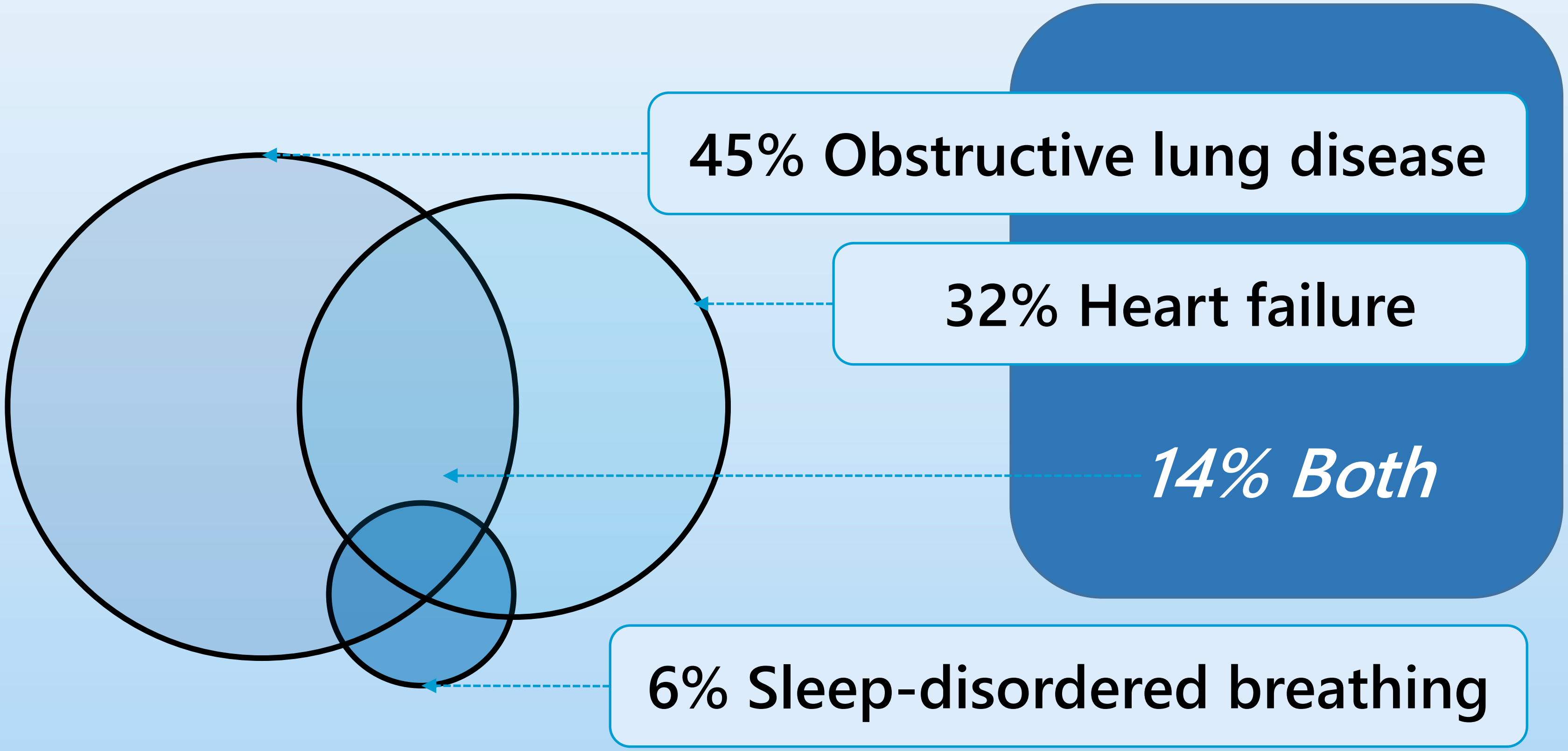

Respirology

Causes of hypercapnic respiratory failure and associated in-hospital mortality
Chung et al. *Respirology* 2022; DOI: 10.1111/resp.14388
